# Screening of Schistosomiasis, Strongyloidiasis and Sexually Transmitted Infections in Nigerian Female Sex Workers Living in Rome

**DOI:** 10.3390/pathogens12020274

**Published:** 2023-02-07

**Authors:** Rosalia Marrone, Clarisse Merline Mekombi, Adela Baraghin, Bezualem Yigezu Borecha, Francesca Perandin, Andrea Ragusa, Dorothy Ukegbu Ashamole, Concetta Mirisola, Mbiye Diku

**Affiliations:** 1 UOC Prevenzione Sanitaria, National Institute for Health, Migration and Poverty (INMP), 00153 Rome, Italy; 2 Department of Infectious–Tropical Disease and Microbiology, Istituto di Ricovero e Cura a Carattere Scientifico (IRCCS), Sacro Cuore-Don Calabria Hospital, 37024 Verona, Italy

**Keywords:** FSWs, schistosomiasis, strongyloidiasis, STIs, migrants, health access barriers, helminth infections

## Abstract

Background: Female Sex Workers (FSWs) are at high risk for acquisition and transmission of sexually transmission infections (STIs). Although several studies investigated the diffusion of STIs in this population, none of them investigated the occurrence of helminth infections in FSW coming from endemic regions. This study aims to assess the prevalence of STIs and helminth infections in a cohort of FSWs. Method: authors conducted a prevalent, observational, and descriptive study on 97 Nigerian FSWs aged 17 to 52 years from January to December 2020. Results: a total of 97 FSWs were recruited. Of these, only 82 had completed screening for hepatitis B, C, syphilis, and HIV, while all 97 were screened for schistosomiasis and strongyloidiasis. The prevalence of STIs among FSWs in Rome was lower than in other European countries. The overall prevalence of HIV and HBsAg were 1.2%, (1/82) and 2.4% (2/82), respectively, while no case of hepatitis C and syphilis was found. Regarding parasitological screening, the overall prevalence of schistosoma species was 4.1% (4/97) while 5.15% (5/97) were positive for strongyloidiasis. Conclusions: our study shows a low prevalence of STIs in Nigerian FSWs except for Hepatitis B and a higher prevalence of schistosomiasis and strongyloidiasis. The permanent monitoring of STI and parasitic infections in sex workers coming from Africa is strongly warranted, especially for hepatitis B, schistosomiasis and strongyloidiasis, to allow a timely diagnosis and treatment, and to plan preventive strategies.

## 1. Introduction

In Italy, the number of Nigerian migrant women carrying out prostitution activities has increased in the last years, with important health implications [1,2]. According to the report of the International Organization for Migration (IOM) [3], 55% of women in prostitution in Italy are foreign-born and 36% of them come from Nigeria. IOM maintains that about 80% of Nigerian female migrants who arrived by sea in 2016 ended up being exploited as sexual slaves. The victims are very young girls, mostly minors, coming from different regions of Nigeria: Edo, Delta, Lagos, Ogun, Anambra, Imo. Different studies have shown that sex workers may undergo vulnerability for several health issues, including those related to mental and sexual health which, in this population, are exacerbated by the experience of discrimination and stigma, resulting in reduced access to health services [4]. Additionally, the high rates of alcohol abuse [5] and experiences of violence as reported in the literature [6] contribute to the vulnerability of this group of women.

From the point of view of infectious diseases, several studies have investigated the prevalence of sexual infections in this group of women. However, although neglected tropical diseases such as schistosomiasis and strongyloidiasis are common in their countries of origin, no study to the best knowledge has investigated the presence of these parasitic infections in this group. The timely diagnosis and treatment of STIs in FSW are important in order to understand the prevalence of these infections and plan appropriate prevention and control strategies. Instead, the diagnosing and treating of strongyloidiasis and schistosomiasis offer the opportunity to avoid serious chronic complications in those with the infection and need to be considered a priority, even if they are causing no harm to the autochthonous population.

This study describes the prevalence of hepatitis B (HBV), hepatitis C (HCV), syphilis, human immunodeficiency virus (HIV) and schistosomiasis and strongyloidiasis infection in Nigerian Female Sex Workers attending the National Institute for Health, Migration and Poverty (INMP) in Rome, according to the Italian [7] and ECDC guidelines [8].

## 2. Materials and Methods

### 2.1. Ethical Aspects 

The study was approved by the Ethical committee of the Italian Higher Istitute of Heatlh (Istituto Superiore di Sanità –PRE-712/16)on 30 July 2019.

### 2.2. Type of Study

Longitudinal, prevalence study.

### 2.3. Study Population

The present study was carried out in the INMP gynecological and infectious units, from January 2020 to December 2020. The study population consisted of Nigerian women who were recruited soon after (around three months) their arrival by contacting the local reception centers where they were hosted and proposing training sessions on some gynecological and infectious issues and free medical examinations. All the Nigerian women included in the study underwent a complete gynecological and medical examination. A cultural mediator was also present during the counselling activity in order to facilitate and strengthen the relationship between the FSWs and the health service. When necessary, patients were referred to gynecological or infectious disease departments for further investigation or hospitalization.

### 2.4. Diagnostic Tests

All women registered with the Italian National Health System were tested for hepatitis B surface antigen (HBsAg), hepatitis B surface antibody (anti-HBs), hepatitis B core antibody (HBcAb).

Antibodies and anti-hepatitis C virus (HCV) antibodies. All assays for HBV and HCV were detected using ELISA (Beckman Coulter, Inc, Fullerton, CA, USA). Anti-HBs and anti-HBc antibodies were detected using commercial immunoassay methods following the manufacturer protocols. HBsAg was detected via an electrochemiluminescence immunoassay (ECLIA) and anti-HCV antibodies via an enzyme-linked immunosorbent assay (ELISA). Screening for syphilis was performed via a reverse syphilis test algorithm, which starts with an assay to measure specific IgM and IgG antibodies with the *Treponema pallidum* (*T. pallidum*) (TPA). If the TPA screen was reactive, samples were tested for rapid plasma reagin to assess disease activity. If this test was negative, the sample was tested for a second *T. pallidum*-specific test—the *T. pallidum* particle agglutination, TP-PA—to confirm the initial TPA screen. Screening for HIV infection was performed using an ELISA (Beckman Coulter, Inc.), and a Western blot (Fujirebio Diagnostics) was used as confirmatory test. 

Serological tests for strongyloidiasis:IFAT diagnostic procedure is an in-house method implemented at the IRCCS Sacro Cuore Don Calabria hospital. It detects IgG antibodies against *S. stercoralis* for antigen preparation, intact *S. stercoralis* filariform larvae are obtained from a positive charcoal fecal culture. A positive result is defined as a titre ≥ 1:80. Bordier ELISA (Bordier Affinity Products, Lausanne, Switzerland) detects Strongyloides IgG antibodies by using somatic antigens from the larvae of *Strongyloides ratti*. The test was performed as per the manufacturer’s instructions. However, as the cut-off varies between runs, we use a normalized optical density (OD) ratio to compare the results obtained in different sessions. A ratio ≥ 1 defines positive results.

Serological tests for schistosomiasis: Bordier ELISA (*Schistosoma mansoni* ELISA kit, Bordier Affinity Products SA, Crissier, Switzerland) detects Schistosoma IgG antibodies by using antigens from an adult of *S. mansoni*. The test was performed as per the manufacturer’s instruction. In order to be able to compare results from different runs, we defined as positive samples those with: the optical density (OD) of the study sample/OD of weak positive serum ≥ 1 (normalized OD). Schisto II Western Blot IgG. (SCHISTO II WB IgG test LDBIO Diagnostics, Lyon, France) The test strip is able to detect *S. mansoni + S. haematobium* antigen strips. The test was performed as per the manufacturer’s instructions using a semi-automated instrument (Dynablot, DYNEX Technologies, Buštěhrad, Czech Republic). Schistosoma ICT IgG-IgM (LDBIO Diagnostics, Lyon, France). This immune-chromatographic test (ICT) was carried out according to the manufacturer’s instructions. The tests were considered as positive or negative depending on whether or not a colored band had appeared.

Urine microscopy: the urine was shaken and filtered through a 25-mm diameter small meshed filter (12 μm Nucleopore), and finally placed on a labeled slide and examined under a microscope (100×) for the detection of Schistosoma eggs.

Stool microscopy: a single stool sample per patient was fixed in 4% formalin, submitted to the formol–ether concentration and examined (100× magnification).

DNA detection using RT-PCR from faeces: a single stool sample per patient was collected in 99.8% ethanol. The DNA extraction and RT-PCR analysis was performed as previously described by Formenti et al. [9]. 

DNA detection using RT-PCR from urine: a single urine sample was obtained (from 10 a.m. to 12 a.m.) for each patient. DNA extraction and RT-PCR were performed as previously described. For the DNA extraction, we followed the protocol published by Pomari et al. [10]. 

All biological samples were collected at the INMP. Aliquots were sent to the laboratory of San Camillo hospital, Rome, for the full blood count and HIV, HBV, HCV, syphilis screening. Other samples were sent to the Department of Infectious and Tropical Diseases and Microbiology, IRCCS Sacro Cuore Don Calabria Hospital in Negrar, Verona, to perform the helminthes screening. All positive individuals at any screening test were referred to the INMP specialists on infectious diseases for the appropriate clinical management, and active immunization was offered to women who were susceptible to HBV infection.

The results were then anonymously entered in an Excel database.

## 3. Results

From January to December 2020, 97 FSWs, aged 17 to 52 years, were recruited. Their period of stay in Italy was less than 3 months and they had been practicing prostitution for about 3–6 months. The mean age of onset of sexual activity was 20 years. No women reported using drugs. All 97 women were screened for *Schistosoma* spp. and *S. stercoralis* whereas only 82 out of 97 (84.5%) were screened for hepatitis B, C, syphilis and HIV, due to the lack of an Italian health card. The overall seroprevalence of *Schistosoma* spp. was 4.1% (4/97), while 5.1% (5/97) were positive for strongyloidiasis. No fecal or urine tests were positive for schistosoma, whereas one fecal molecular test was positive for strongyloidiasis. In only one case was a woman positive for both helminth infections.

Results regarding schistosomiasis and strongyloidiasis screening using different tests are shown in Table 1. 

Regarding the STI screening, none had been previously screened either in their countries or in Italy. One woman was HIV positive and two were positive for HBsAg, while none were positive for hepatitis C or syphilis. Regarding screening for hepatitis B: eight patients (9.7%) had had a previous infection (anti-HBs and -HBc positive) and five (6%) were vaccinated, of whom all had a protective antibody titer. STI screening results are shown in Table 2.

## 4. Discussion

In our cohort of eighty-two screened FSWs, one (1.2%) was positive for HIV, two (2.4%) were HBsAg positive and eight (9.7%) had had previous episodes of HBV infection. Of the 82 women screened for STIs none were positive for syphilis or hepatitis C. Compared to other series published in the literature [2,11], we found a lower prevalence of STIs in our sample. A systematic review by Platt et al. [10] had found a higher prevalence of STIs with a higher prevalence of HIV infection in FSWs from Africa than FSWs from other regions, in accordance with the high prevalence of HIV/AIDS in Africa. FSW remain at risk for HIV; however, in our population the short duration of the sex work did not allow us to measure such risk. Data from an Italian study [12] on African FSWs showed a 3.5% prevalence of HBsAg, and a prevalence of HBcAb in 44%. Another study [13] showed a high prevalence of hepatitis B virus among FSWs in Nigeria compared to other groups, demonstrating that active sexual transmission is an important factor in the spread of HBV in this country where HBV is endemic, and that sex workers have a crucial role in maintaining and transmitting the virus. However, in the literature, variability in the prevalence of HBsAg and STI among Nigerian prostitutes do exist [11,14]: in particular, in another Italian study conducted by Prestileo et al. [15], none of the Nigerian prostitutes screened tested positive for HBsAg, Hepatitis C or syphilis. The lower prevalence of hepatitis B and C in our population may have several explanations: the young age of our patients; the relatively short period of prostitutionand the delay of sexual initiation compared to other studies. Moreover, the girls lived in reception centers where they received condoms and information about safe sex. In our study, the rate of hepatitis B vaccination among immigrate women is also very low (6%), so dedicated programs should be implemented. Furthermore, there was no drug addiction among the screened population. In accordance with other studies [16], sexual transmission is not a primary route of transmission for HCV infection. In addition, while hepatitis B is generally acquired in the countries of origin of immigrants from sub-Saharan Africa, where there is a high prevalence of HBV, HCV is generally less prevalent in Sub-Saharan Africa, being more prevalent in western Europe [17,18].

Studies conducted in Africa showed a low prevalence of syphilis in West Africa [19].

Moreover, the women in our study had been present in Italy for a short time and did not have their own partner. Some studies have shown that FSWs have a higher risk of contracting STIs from their non-paying sexual partners than from their clients [20,21].

Nevertheless, our screening, as well as other studies carried out on immigrants in general [22,23], show a higher seroprevalence of schistosomiasis (4.1%) and strongyloidiasis (5.1%) also in asymptomatic individuals.

In agreement with other researchers [24], we observed that seroprevalence findings were higher than stool-based figures for both parasites, supporting the use of serological screening because of their higher sensitivity, as indicated by Italian and European guidelines [7,8]. 

Direct methods have a specificity of 100% but their sensitivity varies with the prevalence and intensity of infection, as well as with the number of specimens collected and slides prepared for microscopy, stool consistency and circadian and day to day variation of egg counts in stool and/or urine [25,26].

A diagnosis of schistosomiasis via the detection of specific antibodies is more sensitive than microscopy, particularly in light infections [26]. The lower likelihood of a positive direct stool examination in light intensity infections is a common occurrence in the context of screening, and also likely contributed to the poor stool detection rates in our study. 

Regarding the stool and urine real-time polymerase chain reaction (PCR), some studies [27,28] have shown that the accuracy of the stool and urine PCR was similar to microscopy, indicating that this method also lacks sensitivity.

In our study, due to logistical problems of migrants who often lived far from our hospital and the cultural barriers that made them reluctant to deliver stool samples due, only one stool and urine sample was collected per patient, and this too can also explain suboptimal results. 

Regarding the diagnosis ofstrongyloidiasis, the microscopy stool examination has an extremely low sensitivity for *S*. *stercoralis*, and even if stool PCR are more sensitive than stool microscopy, the faecal polymerase-chain reaction sensitivity is still unsatisfactory [29]. So far, serology is the method that also demonstrated the best sensitivity in the diagnosis of strongyloidiasis.

However, although antibody tests do not distinguish between past and current infection, serology is useful for identifying asymptomatic people who may have been exposed and may benefit from treatment.

Infact screening for schistosomiasis and strongyloidiasis is critical since these infections can potentially cause chronic health problems and serious consequences with high risk of potentially fatal complications [30].

Strongyloidiasis is a soil-transmitted helminth infection, with the ability to persist and replicate within a human host for many years as a result of an autoinfection cycle. Under immunosuppressant conditions (such as corticosteroid therapy, organ transplantation and human T-lymphotropic virus 1 infection) it may lead to severe clinical manifestations, such as hyperinfection syndrome and disseminated strongyloidiasis [31].

Schistosomiasis is an infection transmitted through freshwater exposure from the ova shed via the stools (*S. mansoni*) or urine (*S. haematobium*) of infected hosts. Potentially serious long-term complications result from the host immune response to the schistosome eggs: *S. mansoni* can cause fibrosis of the liver, while *S. haematobium* is associated with bladder neoplasia [32].

Unfortunately, the delayed diagnosis of strongyloidiasis and schistosomiasis, often due to a lack of clinical suspicion [33,34], nonspecific clinical presentation, and the use of inappropriate, poorly sensitive diagnostic tests, has long caused an underestimation of the actual global burden of both infections [35,36].

## 5. Limitation of this Study

The small number of women enrolled is a limit of this study. Due to operational reasons, the recruitment lasted only 6 months. Moreover, it is particularly difficult to reach the population [2,11]. Our study may not reflect the overall prevalence of STIs in Nigerian immigrants engaged in prostitution for the following reasons: we recruited women hosted in reception centers where they could already receive information on STIs and free condoms; all women subjected to screening recently arrived in Italy and were forced into prostitution for a short time; no one was a drug user. Last, unfortunately not all the women included in the study could perform all the STIs screening tests due to a lack of health insurance.

## 6. Conclusions

Our study strongly warrants a permanent monitoring of STI and parasitic infections in sex workers coming from Africa, according to the Italian and European guidelines [7,8], in order to allow a timely diagnosis and the appropriate treatment, and to plan preventive strategies in this vulnerable and marginalized population. In particular, we stress the importance of schistosomiasis and strongyloidiasis screening, two neglected diseases often underestimated due to their asymptomatic or paucisymptomatic expression in the chronic phase, but which have considerable clinical and public health impacts [23].

At the same time, providing training sessions and guidance activities for this population is essential, considering that many patients were unable to perform the exams because they did not have the Italian health card, albeit being legally entitled to get it.

In order to implement and improve screening and counselling activities for these women and to ensure adequate access to health services, it is necessary to consider barriers to healthcare access often related to linguistic and cultural issues, as well as concerns related to their status of undocumented immigrants.

Prevention programs should take into account all social and cultural factors in order to improve their trust in public health services.

According to our experience, we believe that STI prevention and control are crucial for involving FSWs in specific educational programs with a multidisciplinary group of professionals (gynecologist, infectivologist, psychologists, transcultural mediators, lawyers, nurses, anthropologists, social workers), to improve their awareness about risk behavior, and provide all the necessary support. A cultural mediator is always present during counselling activities in our clinical settings, in order to facilitate and strengthen the relationship between the overall health system and the patient, as well as to focus on STI acquisition risk and the importance of promoting safe sex. Indeed, we have found that most women had an insufficient knowledge of the risk of STI acquisition and its prevention before they are informed in the centers about them and the practice of safer sex.

Ultimately, our study shows how thee early identification and reception of trafficked women in centers networked with health services is important for their health and access to health facilities, the prevention of infections and timely care in case of illness. Good reception breeds good health!

## Figures and Tables

**Table 1:** Schistosomiasis and strongyloidiasis screening methods.

PARASITE	ELISA Pos	WB IgG Pos	Urine PCR	Urine Microscopy	Stool PCR	Stool Micrcopy
*Schistosoma* spp. n 97	4/97 (4.1%)	4/97 (4.1%)	0/97	0/97	0/97	0/97
*Strongyloides stercoralis* n97	5/97 (5.1%)	-	-	-	1/97(1%)	0/97

**Table 2:** STI screening results.

Patients n 82	Anti-HIV	HBsAg+	Anti HCV	Syphilis Serology
	1/82 (1.2%)	2/82(2.4%)	0/82	0/82

## Data Availability

The data that support the findings of this study are available from the corresponding author upon reasonable request.
